# Supplementary material for: Core Fucosylation Mediated by the FucT-8 Enzyme Affects TRAIL-Induced Apoptosis and Sensitivity to Chemotherapy in Human SW480 and SW620 Colorectal Cancer Cells
Source: Int J Mol Sci. 2023 Jul 25;24(15):11879. doi: 10.3390/ijms241511879 (PMC10418920; doi:10.3390/ijms241511879)
Supplement: Supplementary file 1 [file ijms-24-11879-s001.zip › ijms-2499045-supplementary.pdf]

Supplementary material

Table S1: Primer sequences for real-time quantitative PCR (RT-qPCR).

Gene ID	Gene name	Accession number	Assay ID
FUT8	Fucosyltransferase 8	NM_004480.4	Hs00189535_m1
GAPDH	Glyceraldehyde-3-phosphate dehydrogenase	NM_002046	Hs99999905_m1
ABCB1	ATP binding cassette subfamily B member 1	NM_000927.4	Hs00184500_m1

Supplementary Figure S1

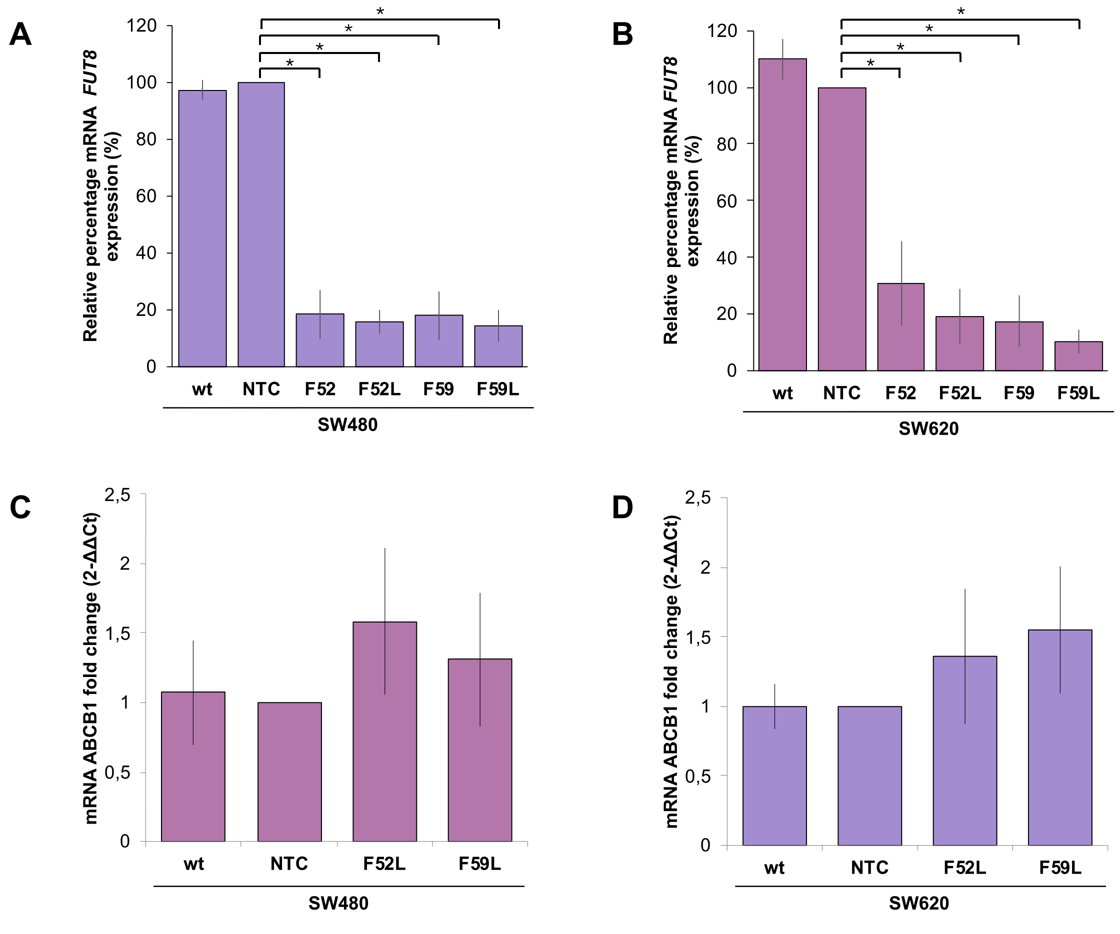

Figure S1: Quantification of the mRNA of the *FUT8* gene after selection with LCA (A-B) and of the ATP binding cassette subfamily B member 1 (*ABDCB1*) gene (C-D) in the SW480 and SW620 lines. The mRNA was extracted from growing mid-passage cells and analysed by real-time quantitative PCR (RT-qPCR). *FUT8* and *ABDCB1* mRNAs were quantified relative to that of the housekeeping gene *GAPDH*. Relative expression between cell groups was

determined using the $2^{-\Delta\Delta Ct}$ fold-change method. Measurements were obtained from three different experiments, and results were plotted as the mean ± SD. For statistical calculations, the NTC clone was used as the control cell line. One-way ANOVA test results were significant, and multiple comparisons between the groups were carried out using Fisher's multiple-comparison test. Results were considered significant at (*) $p < 0.05$. wt: wild-type cells; NTC: non-targeted control cells; F52L and F59L: *FUT8*-knockdown clones selected with *Lens culinaris* agglutinin (LCA).

Supplementary Figure S2

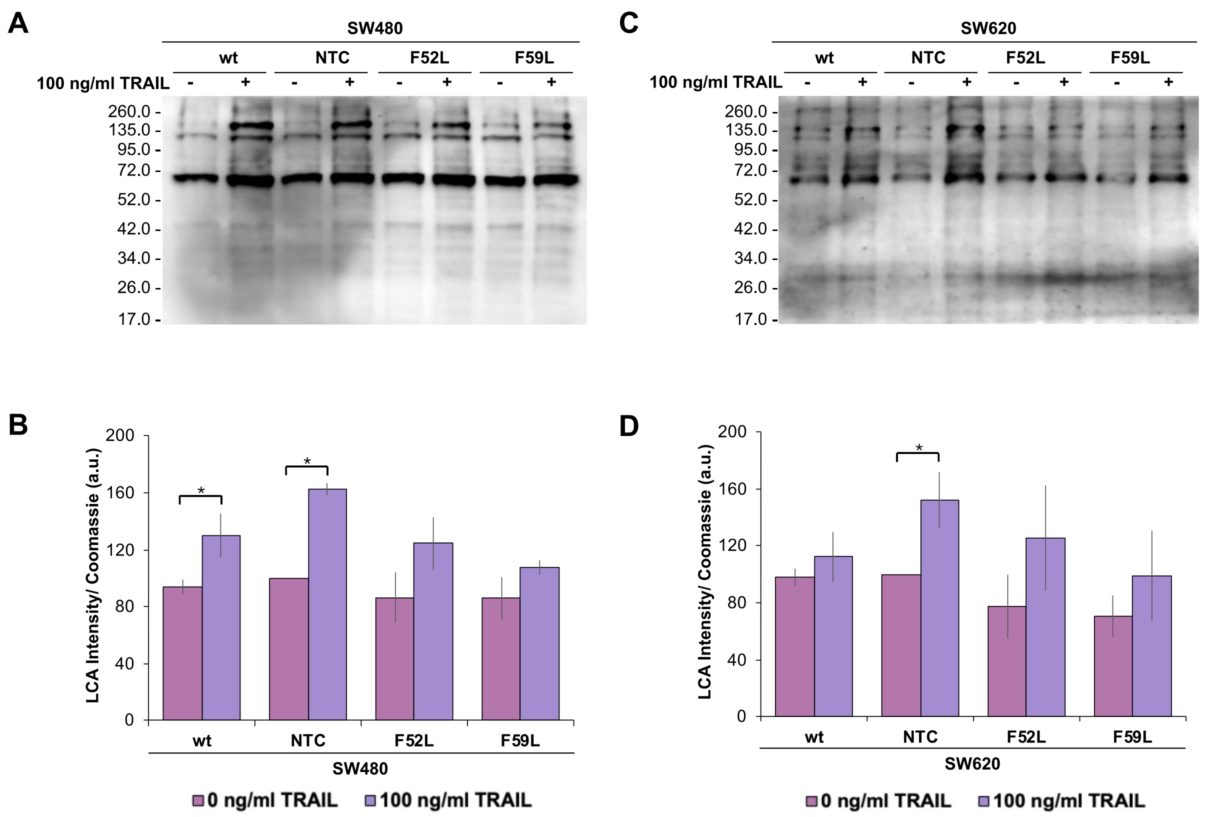

Figure S2: Quantification of fucosylated proteins by chemiluminescence using biotinylated *Lens culinaris* agglutinin (LCA) as the detection lectin in SW480 (A,B) and SW620 (C,D) cells. The protein loading control was verified after dyeing the PVDF membranes with Coomassie R-250. Measurements were taken from three different experiments. Results are plotted as the mean ± SD. For statistical calculations, NTC clones were used as

reference. The Mann–Whitney U test results were significant at $p < 0.05$ (*). wt: wild-type cells; NTC: non-targeted control cells; F52L and F59L: *FUT8*-knockdown clones selected with *Lens culinaris* agglutinin (LCA).

Supplementary Figure S3

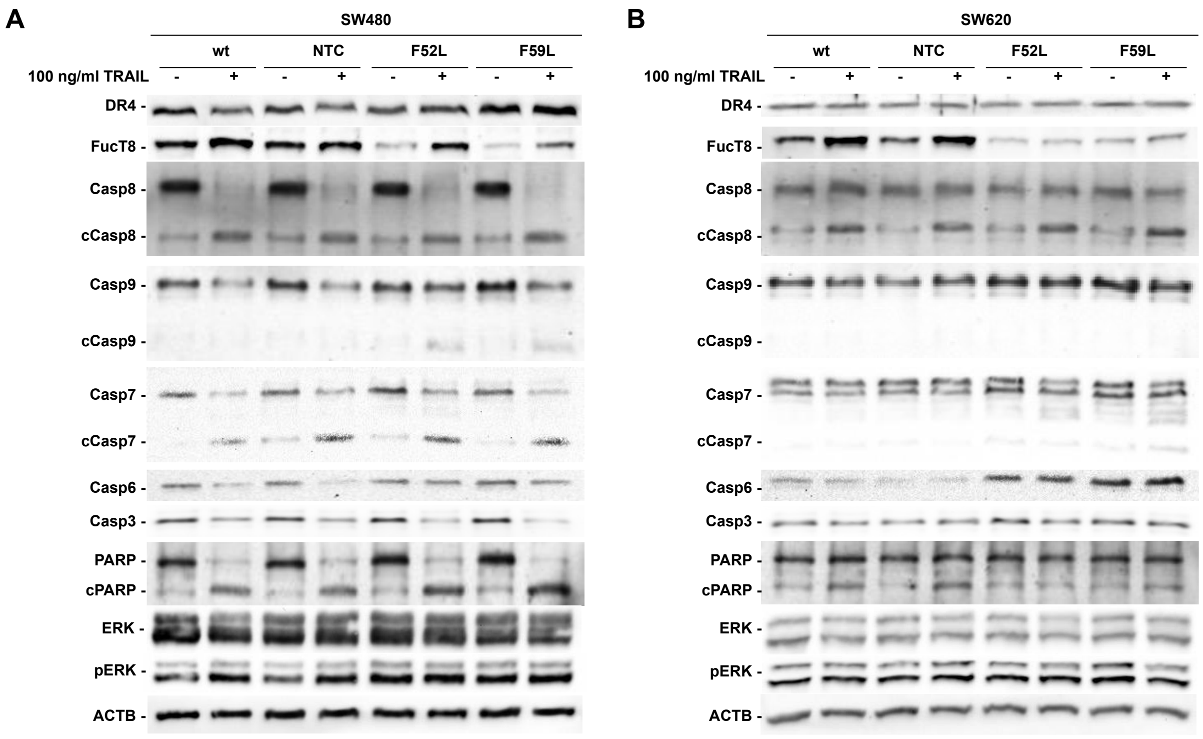

Figure S3: Representative immunoblots of DR4, FucT-8, pro-caspase-8 (Casp8), caspase-8 (cCasp8), pro-caspase-9 (Casp9), caspase-9 (cCasp9), pro-caspase-7 (Casp7), caspase-7 (cCasp7), pro-caspase-6 (Casp6), pro-caspase-3 (Casp3), PARP, cleaved PARP (cPARP), total ERK1/2 and phosphorylated ERK1/2 (pERK) in SW480 (A) and SW620 (B) lines. Cells were maintained either in normal medium (-) or in medium supplemented with 100 ng/mL TRAIL for 24 h (+). β-actin expression (ACTB) was used as the protein loading control. wt: wild-type cells; NTC: non-targeted control cells; F52L and F59L: *FUT8*-knockdown clones selected with *Lens culinaris* agglutinin (LCA).
